# Supplementary material for: Preparation and Properties of Cyclodextrin Inclusion Complexes of Hyperoside
Source: Molecules. 2022 Apr 25;27(9):2761. doi: 10.3390/molecules27092761 (PMC9100073; doi:10.3390/molecules27092761)

Supplement information

Preparation and properties of cyclodextrin inclusion complexes of *Hyperoside*

Xinyu Zhang†, Jianqing Su*†, Xiaoya Wang, Xueyan Wang, Ruixue Liu, Xiang Fu, Ying Li, Jiaojiao Xue, Xiaoli Li, Rui Zhang, Liuling Chu*

College of Agronomy and Agricultural Sciences, Liaocheng University, Liaocheng 252000, China

*Corresponding author: sujianqing@lcu.edu.cn (J.S.); chuxiul@163.com (X.C.);
Tel.: +86-150-9503-9358 (J.S.); +86-150-2062-6235 (X.C.)

† There authors have contributed equally to this work.

Figure S1

^{1}H NMR spectra of Hyp, 2H-β-CD, and Hyp–2H-β-CD.

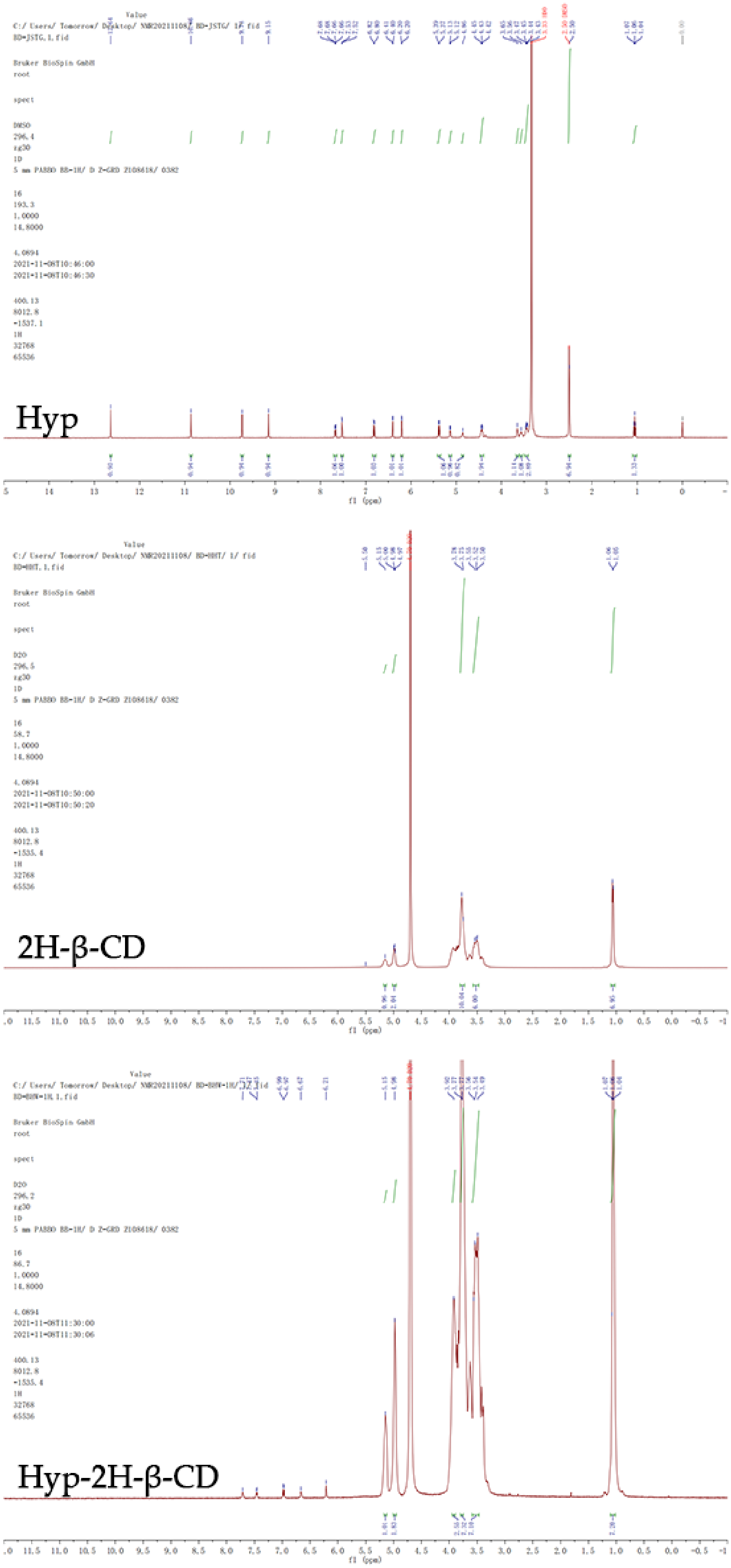

Supplement: Supplementary file 1 [file molecules-27-02761-s001.zip › molecules-1669537-supplementary.pdf]
